# Cerebral blood flow impairment and cognitive decline in heart failure

**DOI:** 10.1002/brb3.2176

**Published:** 2021-05-14

**Authors:** Ana Ovsenik, Matej Podbregar, Andrej Fabjan

**Affiliations:** ^1^ Faculty of Medicine University of Ljubljana Ljubljana Slovenia; ^2^ Department of Cardiology University Medical Centre Ljubljana Ljubljana Slovenia; ^3^ Faculty of Medicine Department for Internal Medicine University of Ljubljana Ljubljana Slovenia; ^4^ Department of Intensive care General Hospital Celje Celje Slovenia; ^5^ Faculty of Medicine Institute for Physiology University of Ljubljana Ljubljana Slovenia; ^6^ Department of Vascular Neurology and Intensive Care Neurological Clinic University Medical Centre Ljubljana Ljubljana Slovenia

**Keywords:** autonomic nervous system, cerebral autoregulation, cerebral blood flow, cognitive decline, heart failure, neurovascular coupling

## Abstract

**Background and Purpose:**

Cognitive decline is an important contributor to disability in patients with chronic heart failure, affecting 25%–50% of patients. The aim of this review is to stress the importance of understanding pathophysiological mechanisms of heart failure involved in cognitive decline.

**Methods:**

An extensive PubMed search was conducted for the literature on the basic mechanisms of cerebral blood flow regulation, the effect of cardiac dysfunction on cerebral blood flow, and possible mechanisms underlying the association between cardiac dysfunction and cognitive decline.

**Results:**

Published literature supports the thesis that cardiac dysfunction leads to cerebral blood flow impairment and predisposes to cognitive decline. One of the postulated mechanisms underlying cognitive decline in chronic heart failure is chronic regional hypoperfusion of critical brain areas. Cognitive function may be further compromised by microvascular damage due to cardiovascular risk factors. Furthermore, it is implied that cerebral blood flow assessment could enable early recognition of patients at risk and help guide appropriate therapeutic strategies.

**Conclusion:**

Interdisciplinary knowledge in the fields of neurology and cardiology is essential to clarify heart and brain interconnections in chronic heart failure. Understanding and identifying the basic neuropathophysiological changes in chronic heart failure could help with developing methods for early recognition of patients at risk, followed by institution of therapeutic actions to prevent or decrease cognitive decline.

## Graphical abstract

One of the postulated mechanisms underlying cognitive decline in chronic heart failure is chronic regional hypoperfusion of critical brain areas. Cognitive function may be further compromised by microvascular damage due to cardiovascular risk factors in chronic heart failure patients. Cerebral blood flow assessment is proposed for early recognition of patients at risk of cognitive decline and could guide further therapeutic strategy.

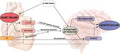

## 1 INTRODUCTION

Although the human brain represents only 2% of adult body mass, it requires 20% of resting metabolic rate (Phillips et al., 2016). With almost no energy stores, this high cerebral energy demand comes at a cost of a constant supply of metabolites. Unsurprisingly, the brain receives 15% of the total cardiac output and is highly reliant on the proper functioning of the heart (Willie and Smith, 2011). The catastrophic consequence of sudden cessation of blood flow to the brain manifests as stroke; however, subtler cerebral blood flow (CBF) alterations can cause chronic brain injury in vulnerable areas, which can lead to cognitive decline (CD) (Iadecola, 2017). CD represents one of the main contributors to disability and loss of autonomy in patients with chronic heart failure (CHF), affecting 25%–50% of patients (Pressler, 2008). CHF is related to increasing age, which is also associated with CD (Tarumi & Zhang, 2018). To what extent the risk factors in aging population on one hand and cardiac dysfunction on the other contribute to CD is still unknown. Interdisciplinary knowledge in neurology and cardiology may clarify this causative relationship. Therefore, we aim to stress the importance of understanding the pathophysiological mechanisms of CHF involved in CD. In this review of the literature, we discuss (i) cerebrovascular tree structure relevant for understanding CBF regulation, (ii) basic mechanisms of CBF regulation, (iii) effect of cardiac dysfunction on CBF, (iv) possible mechanisms underlying the association between cardiac dysfunction and CD, (v) and potential role of CBF evaluation in CHF patients.

## 2 CEREBROVASCULAR TREE STRUCTURE

**FIGURE 1:**
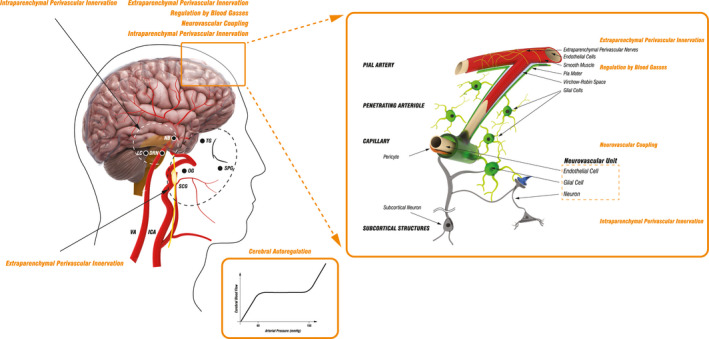
Schematic presentation of cerebrovascular tree structure and cerebral blood flow regulatory mechanisms. The brain receives arterial perfusion through two internal carotid arteries (ICA) and two vertebral arteries (VA) that interconnect intracranially through the circle of Willis, which gives rise to several vessels branching into progressively smaller arteries that run along the surface of the brain and then penetrate the brain tissue continuing deeper into the brain until they terminate in a network of capillaries. The main mechanisms regulating cerebral blood flow (CBF) include cerebral autoregulation (CA), activity of perivascular nerves, regulation by blood gasses, and neurovascular coupling. Cerebral autoregulation (CA) ensures CBF is maintained approximately constant across a wide range of mean arterial pressure (MAP), with neck vessels and large cerebral arteries serving as the first line of defense against hyperperfusion of the downstream microvasculature. CBF is highly dependent on extraparenchymal and intraparenchymal perivascular innervation. Extraparenchymal cerebral blood vessels are extensively innervated by autonomic and sensory nerve fibers deriving from the superior cervical ganglion (SCG), sphenopalatine ganglion (SPG), otic ganglion (OG), and the trigeminal ganglion (TG), while intraparenchymal vessels receive innervation through projections from subcortical neurons originating in locus coeruleus (LC), nucleus basalis (NB), and dorsal raphe nucleus (DRN). Pial arterioles are considered to be the main site of resistance regulation associated with changes in the partial pressure of blood gasses and of pH. Neurovascular coupling, which defines the association between neurons, microvascular endothelial cells, and glial cells, initiates local CBF changes on the level of capillaries. This signal is then transmitted upstream to remote vessels, including pial arterioles, which leads to broader blood flow changes. Additionally, neurovascular coupling may be modulated through subcortical neuronal activity. Internal carotid artery (ICA); vertebral artery (VA); superior cervical ganglion (SCG), otic ganglion (OT); sphenopalatine ganglion (SPG); trigeminal ganglion (TG); superior cervical ganglion (SCG); locus coeruleus (LC); dorsal raphe nucleus (DRN); nucleus basalis (NB)

The brain receives arterial perfusion through two pairs of large arteries. The internal carotid arteries supply the anterior portion of the cerebrum, while vertebral arteries supply the posterior part of the cerebrum, cerebellum, and brainstem (Figure 1). The circle of Willis, interconnecting the anterior and posterior cerebral circulation, gives rise to the paired anterior, middle, and posterior cerebral arteries, which divide into progressively smaller arteries that run along the surface of the brain within the subarachnoid space until they penetrate the brain tissue (Figure 1). The smallest surface vessels, the pial arterioles, give rise to smaller arterioles with a thinner smooth muscle cell layer that penetrate the brain surface, entering the Virchow Robin space (Phillips et al., 2016). The penetrating arterioles are surrounded by pial cells, nerve fibers, macrophages, mastocytes, collagen fibers, and others (Iadecola, 2017). Deeper in the brain, the parenchymal arterioles, which have only a single layer of smooth muscle cells, are enclosed by astrocytic end‐feet and in places surrounded by neural processes projecting from subcortical structures. Parenchymal arterioles continue into a network of capillaries, in which smooth muscle cells are replaced by pericytes. The surrounding astrocytic end‐feet and neural processes projecting from subcortical structures remain in close contact with capillaries (Iadecola, 2017). With penetration of arterioles deeper in the brain tissue, the density of blood vessels decreases. Moreover, after traversing cortical gray in a straight line, they begin to coil, loop, and spiral when they reach the subcortical white matter (Nonaka et al., 2003). Due to this, subcortical areas are more prone to ischemic damage than superficial gray matter when global CBF is compromised under the influence of adverse systemic factors.

## 3 BASIC MECHANISMS OF CBF REGULATION

Multiple regulatory mechanisms overlap to provide tight CBF control due to the brain's high metabolic demand for oxygen, limited intracellular capacity for energy storage, rapid changes of metabolic demand with neuronal activity, and limited space of enclosed cranium (Toth et al., 2017). It has been shown repeatedly that the most influential factors determining CBF are partial pressure of arterial carbon dioxide (PaCO_2_), mean arterial pressure (MAP), rate of cerebral metabolism, and activity of perivascular nerves (Figure 1). Disruption in any of the regulatory mechanisms can lead to CBF disturbance.

### 3.1 Regulation by blood gasses

Cerebral vessels, including the large arteries of the neck, large intracranial arteries, pial arterioles, and parenchymal vessels, possess the unique property of high sensitivity to PaCO_2_ (Willie et al., 2012). With their anatomical position in the subarachnoid space, surrounded by cerebrospinal fluid and exposed to local metabolic conditions, pial arterioles are believed to be the main site of resistance regulation due to changes in the partial pressure of blood gasses (Figure 1) (Willie et al., 2014). A 1‐mmHg change above or below eupnoeic PaCO_2_ leads to an approximate 3%–6% increase or a 1%–3% decrease in flow, respectively (Sato et al., 2012; Willie et al., 2012). The sensitivity to hypoxia is, however, less strong, with changes affecting CBF only when partial arterial O_2_ pressure falls below 50 mmHg. Hypoxia induces a 0.5%–2.5% increase in CBF when arterial O_2_ saturation falls by 1% (Willie et al., 2014).

Increased PaCO_2_ in arterial blood causes changes of cerebrovascular resistance independently of arterial pH (Lassen, 1968). CO_2_ molecules are believed to cross the cerebrovascular blood–brain barrier and induce a change in pH in the extracellular space of the vessel, which alters vascular smooth muscle tone (Lassen, 1968). However, the mechanism of vasodilatation in case of hypoxia is poorly defined. Suggested mechanisms for CBF change include hypoxia‐related extracellular acidosis, hypoxia‐related signals arising from neurovascular unit, and other mediators present in hypoxia (adenosine and nitric oxide) that directly elicit vasodilatory response (Willie et al., 2014).

### 3.2 Cerebral autoregulation

Traditional knowledge on cerebral autoregulation mechanism is based on the concept by Lassen et al., who state that cerebral autoregulation ensures CBF is maintained approximately constant across a wide range of MAP (60–150 mmHg; Figure 1) (Lassen, 1959). CBF represents the quotient between cerebral perfusion pressure (CPP) and cerebrovascular resistance (CVR), while CPP stands for the difference between MAP and intracranial pressure (ICP), summarized by the equation CBF = (MAP–ICP)/CVR. With changes in MAP, CVR changes in order to achieve constant CBF. However, Willie et al. have recently published a contradictive finding suggesting that CBF is much more pressure‐passive than is generally believed and has more efficacious buffering capacity against increases than against decreases in CPP (Willie et al., 2014). Vessel changes due to cerebral autoregulation are most likely mediated by an interplay between myogenic mechanisms, where vessel constricts or dilates in response to changes in blood flow causing shear stress, metabolic factors (including local H+, K+, O_2_, and adenosine concentrations), and local neurogenic mechanisms arising from perivascular autonomic innervation (Paulson et al., 1990). The exact site of CVR modulation in cerebral autoregulation is under debate. The percentage of the total CVR each cerebrovascular segment offers to blood flow reflects their potential to CBF control (Iadecola, 2017). Extraparenchymal vessels are responsible for 60% of the CVR and vessels within the brain for 40% of the CVR (Iadecola, 2017). Therefore, the main effectors of cerebral autoregulation are likely the neck vessels, large cerebral arteries, and the pial arterioles (Willie et al., 2014). Moreover, due to their location, neck vessels and large cerebral arteries can serve as the first line of defense in maintaining CBF and protection of the downstream microvasculature from hyperperfusion (Phillips et al., 2016).

### 3.3 Neurovascular coupling

Neurovascular coupling presents the complex functional association between astrocytes, neurons, and microvasculature, which enables local CBF to adapt to local neuron activity through engagement of the entire cerebrovascular tree, from capillaries deep in the substance of the brain to pial arteries on the brain surface (Figure 1) (Phillips et al., 2016). Current literature proposes capillaries as the site of neural activity detection and further signal transmission to upstream pial arteries (Iadecola, 2017). Neuronal activation leads to increased K+ release, initiating hyperpolarization of the capillary endothelial cells and pericytes, which is transferred to upstream smooth muscle cells via gap junctions and induces vasodilatation (Phillips et al., 2016). Vasodilatation is further increased by the release of local vasoactive substances by neurons and glial cells upon neural activation (nitric oxide, adenosine and adenosine triphosphate, prostanoids, etc.) (Iadecola, 2017). Increased CBF causes shear stress upon the arteriolar wall, which additionally contributes to vasodilatation by myogenic response through the release of vasoactive substances from the arteriolar endothelium (Iadecola, 2017). Thus, the increase in local CBF might be initiated from the direct effect of neural activity on the microvasculature, but the broader regional increase in CBF results from conduction of vasodilatation and myogenic responses to the remote upstream blood vessels.

However, neurovascular coupling also exerts dysfunctional activity in pathological conditions. Subarachnoid hemorrhage can cause inversion of the neurovascular response from vasodilation to vasoconstriction, whereas global ischemia causes a decrease in the magnitude of dilation to the evoked neuronal activity, which can last for days after reperfusion (Koide et al., 2013) The shift from vasodilative to vasoconstrictive neurovascular response can be provoked by several factors, including increased astrocyte end‐foot Ca2+ levels, increased extracellular K+ concentration, and increased end‐foot BK channel activity (Koide et al., 2013) Vasoconstrictive or inverse neurovascular coupling presents a further threat for the already damaged brain after subarachnoid hemorrhage or vulnerable ischemic penumbra.

### 3.4 Perivascular innervation

Extraparenchymal cerebral blood vessels are extensively innervated by perivascular sympathetic, parasympathetic, and sensory nerve fibers, deriving from autonomic nervous ganglia (superior cervical, sphenopalatine and otic ganglion), and the sensory trigeminal ganglion. On the other hand, intraparenchymal vessels are innervated by projections from subcortical neurons originating in locus coeruleus, nucleus basalis, and dorsal raphe nucleus (Figure 1) (Brassard et al., 2017).

Sympathetic postganglionic nerve fibers originate mainly from the superior cervical ganglion, with noradrenaline, neuropeptide Y, and adenosine triphosphate as the main neurotransmitters (Edvinsson, 2011). When activated (e.g. in case of severe hypertension, hypoxia, and hypercapnia), they promote vasoconstriction limiting vasodilatation, modulation of the cerebral autoregulation, and reduction of the ICP and cerebrospinal fluid production (Edvinsson, 2011). Their role in certain pathological conditions, such as hemorrhagic hypotension, might be detrimental (Harper et al., 1972).

Parasympathetic nerve fibers originate from sphenopalatine and otic ganglion and contain acetylcholine, vasoactive intestinal peptide, pituitary adenylate cyclase activating peptide (PACAP), and nitric oxide synthase (NOS) (Edvinsson, 2011). The parasympathetic fibers appear to tonically balance sympathetic vasoconstriction with active vasodilatation, while also dynamically responding to falls in pressure (Hamner et al., 2012). There is some evidence that the parasympathetic nervous system works in concert with trigeminal sensory fibers in certain conditions, such as primary headaches, as mentioned below (Edvinsson, 2002).

Sensory nerve fibers arise from the trigeminal ganglion, terminate with their peripheral axons on the cerebral vessel surface, and project with their central axons onto the second‐order neurons in the trigeminal nucleus caudalis and its caudal extension in the cervical spinal cord (Goadsby et al., 1997). The perivascular sensory nerve fibers contain calcitonin gene‐related peptide (CGRP), substance P (SP), neurokinin A, PACAP, and NOS (Edvinsson, 2011). Involved not only in vascular pain transmission (eg, migraine), but also playing a motor role, the trigeminovascular system is activated in case of threatened CBF limiting excessive vasoconstriction (eg, subarachnoid hemorrhage) or as a neurogenically mediated supplement to cerebral hyperemia after a seizure (Goadsby & Edvinsson, 1993). Stimulation of the elements of trigeminovascular system leads to vasodilatation and increased CBF through a direct (antidromic) and an indirect (orthodromic) way (Goadsby & Edvinsson, 1993). The antidromic way stands for vasodilatation due to the direct effect of vasodilatory substances (CGRP and SP) released from trigeminal peripheral axons upon activation, forming the so‐called trigeminovascular reflex. The orthodromic way, however, includes activation of the trigeminal nerve, traversion of brainstem, and transmission of efferent path signal through parasympathetic fibers of the facial nerve, sphenopalatine ganglion, and otic ganglion, leading to vasodilatation via nicotinic receptors on the cerebral vessel surface.

The most studied subcortical structures that project to cortical microvessels influencing CBF are noradrenergic locus coeruleus, cholinergic nucleus basalis, and serotonergic dorsal raphe nucleus. Noradrenergic fibers originating from locus coeruleus promote vasoconstriction and protect the integrity of the blood‐brain barrier (Harik & McGunigal, 1984). Stimulation of cholinergic fibers leads to vasodilatation, whereas serotonergic fibers promote vasoconstriction or vasodilatation depending on the level of dorsal raphe nucleus stimulation (Hamel, 2006).

## 4 EFFECT OF CARDIAC DYSFUNCTION ON CBF

Chronic heart failure is a systemic disease not limited to the heart itself but affecting other organ systems, including the brain (Kim & Kim, 2015). Although bidirectional heart‐brain interconnection is recognized in the literature, the exact mechanisms underlying it remain a matter of discussion. Studies have assessed cardiac function by measuring left ventricular ejection fraction (LVEF), which represents the percentage of blood volume ejected in one cardiac cycle and cardiac output, the volume of blood pumped by the heart in every minute (Aires et al., 2020). The last is dependent on systolic as well as diastolic properties of the heart and may be calculated from the product of stroke volume, the volume of blood pumped in one heart cycle, and heart rate. The brain is involved in CHF progression through sympathetic activation and regulation of fluid homeostasis, which leads to left ventricular remodeling and symptom worsening (Kim & Kim, 2015). The failing heart with reduced cardiac output, on the other hand, can affect cerebral autoregulation and decrease CBF. Multiple feedback signals promote vicious circle and progression of the disease, which may be interrupted by contemporary treatment options. According to the literature, global CBF is reduced by 14%–30% in severe CHF, significantly increasing after captopril treatment, (Rajagopalan et al., 1984) cardioversion, (Petersen et al., 1989) cardiac resynchronization therapy, (Bommel et al., 2010) and heart transplantation (Gruhn et al., 2001). Moreover, currently used continuous‐flow left ventricular assist devices (CF‐LVADs) have been shown to normalize resting CBF after implantation; however, reduced pulsatility may increase the risk of adverse neurovascular events (Cornwell et al., 2014). Nonpulsatile flow induces microcirculatory changes, such as endothelial dysfunction, reduced nitric oxide bioavailability, and vascular smooth muscle proliferation, which may lead to abnormal cerebral autoregulation predisposing patients to either watershed ischemic strokes or perfusion breakthrough hemorrhagic strokes (Stohr et al., 2019). Furthermore, loss of pulsatility leads to relative hypertension due to high diastolic flow and contributes to the development of cerebral microbleeds, which may be aggravated by acquired von Willebrand factor deficiency caused by shear stress within CF‐LVAD (Frontera, 2020). Recent improvements in outcomes including reduced incidence of stroke can be attributed to the added pulsatility and the greater load sensitivity of the technologically improved CF‐LVADs (Stohr et al., 2019).

The effects of inotropes and inodilators on CBF, frequently used in advanced heart failure management, are mainly based on animal studies, while data in human are scarce. It is generally accepted that catecholamines (including dobutamine) do not cross the blood–brain barrier (BBB) in the mature brain and therefore have minimal influence on the cerebral vasculature and cerebral metabolic rate (Azhan & Wong, 2012). Dobutamine exerts its effects through beta‐1 adrenergic activity leading to increased cardiac output and arterial blood pressure, which would engage cerebral autoregulation to preserve CBF in healthy subjects, while increases in CBF were only found in pathological conditions as in patients after subarachnoid hemorrhage or sepsis (Ogoh et al., 2017). On the other hand, phosphodiesterase inhibitors, known for their inotropic and systemic vasodilating properties, act as direct cerebral vasodilators and are effective in preventing and reversing cerebral vasospasm (Sulek et al., 2002). Likely, levosimendan, a positive inotropic agent with vasodilating properties is known to cross the BBB and may act beneficially in the central nervous system causing arterial vasodilatation and neuroprotection (Farmakis et al., 2016).

The importance of CBF measurement as a promising prognostic tool to identify advanced CHF patients was reported. Kim et al. found that patients with low CBF were nearly 2.5 times more likely to die or require urgent transplantation during a median follow‐up period of 3 years (Kim & Kim, 2015). Furthermore, they concluded also that CBF was associated with recovery of left ventricular systolic dysfunction in patients with idiopathic dilated cardiomyopathy indicating CBF measurement could be helpful to predict the clinical course of CHF (Kim et al., 2012). Choi et al. (2006) found CBF to be related to factors that represent the severity and chronicity of heart failure (NYHA class and pro‐BNP level) while the association with LVEF and exercise capacity was not confirmed. On the contrary, in the study by Loncar et al. (2011) low LVEF and physical performance capacity were the independent determinants of impaired CBF in patients with CHF. This discrepancy may be explained by the selection of patients with more severe CHF, such as those in the study of Choi et al. (2006) where factors other than LVEF contributed more to CBF, including NYHA functional class and neurohormonal system activation (expressed by serum NT‐pro‐BNP levels).

However, global CBF may still be normal in mild CHF due to constantly activated regulatory mechanisms to maintain such normal cerebral perfusion (Erkelens et al., 2017). The reduction in cardiac output in patients with CHF is compensated by dilatation of the brain arterioles, which limits the potential for further dilatation and leads to impaired cerebral autoregulation and cerebrovascular reactivity to CO_2_ (Caldas et al., 2017; Erkelens et al., 2017; Georgiadis et al., 2000; Serber et al., 2014; Xie et al., 2005). More extensive dysfunctional cerebrovascular reactivity predominantly affects the right side of the brain, (Roy et al., 2017; Serber et al., 2014) correlates with worsening of CHF and is found in CHF patients with autonomic nervous system dysfunction (Georgiadis et al., 2000) due to structural and functional changes in areas involved in autonomic nervous system control (Song et al., 2019; Woo et al., 2009).

Additionally, neurohumoral system activation in CHF patients contributes to CBF disruption, provoking vasoconstriction of not only the peripheral vascular beds but also the cerebral vascular bed (Meng et al., 2015). Thus, cerebral resistance vessels change as a result of chronic adaptation to low cardiac output in advanced CHF impairing cerebral autoregulation (Kim & Kim, 2015). With altered cerebral autoregulation, cerebrovascular reactivity to CO_2_, and autonomic nervous system control, everyday activities place the CHF patient at risk for critical decrease of CBF in strategic regions of the brain, leading to loss of consciousness or, in extreme conditions, cerebral ischemia and stroke.

Atrial fibrillation (AF), a condition frequently accompanying CHF, causes CBF disruption through specific mechanisms and will be dealt with separately. It carries a fivefold increased risk for thromboembolic transient ischemic attack and stroke (Gardarsdottir et al., 2018). Moreover, lower total brain, gray and white matter volumes related to poorer cognition, and increased risk of dementia are found in patients with AF (Stefansdottir et al., 2013). The association between AF and decreased brain volume is stronger with increased arrhythmia burden and a longer time since the first diagnosis of AF (Gardarsdottir et al., 2018). It has been recently observed that AF is independently associated with CD through a range of different potential hemodynamic mechanisms including micro‐ and macroembolic events (Gardarsdottir et al., 2018). One of the main postulated mechanisms is, however, reduced CBF in AF due to RR interval variability and loss of atrial systole leading to reduced stroke volume (Saglietto et al., 2019). Porebska et al. (2007) pointed out that medial cerebral artery (MCA) deficit waveforms depicting MCA blood flow, analogous to “pulse deficit” within peripheral circulation during AF attack, may contribute to reduced CBF. The attempt to normalize cardiac output with increasing heart rate is, however, inefficient as tachycardia shortens the ventricular filling time, contributing to even further decrease in stroke volume, cardiac output, and, lastly, even CBF. Furthermore, significant CBF improvement was found in patients after 30 days from cardioversion, if they remained in sinus rhythm (Petersen et al., 1989; Porebska et al., 2007). Patients with paroxysmal AF, but in sinus rhythm during cerebral flow assessment exhibit similar CBF values as those without a history of arrhythmia, supporting the fact that the presence of arrhythmia at the time of imaging is of key importance (Gardarsdottir et al., 2018).

## 5 POSSIBLE MECHANISMS UNDERLYING THE ASSOCIATION BETWEEN CARDIAC DYSFUNCTION AND CD

**FIGURE 2:**
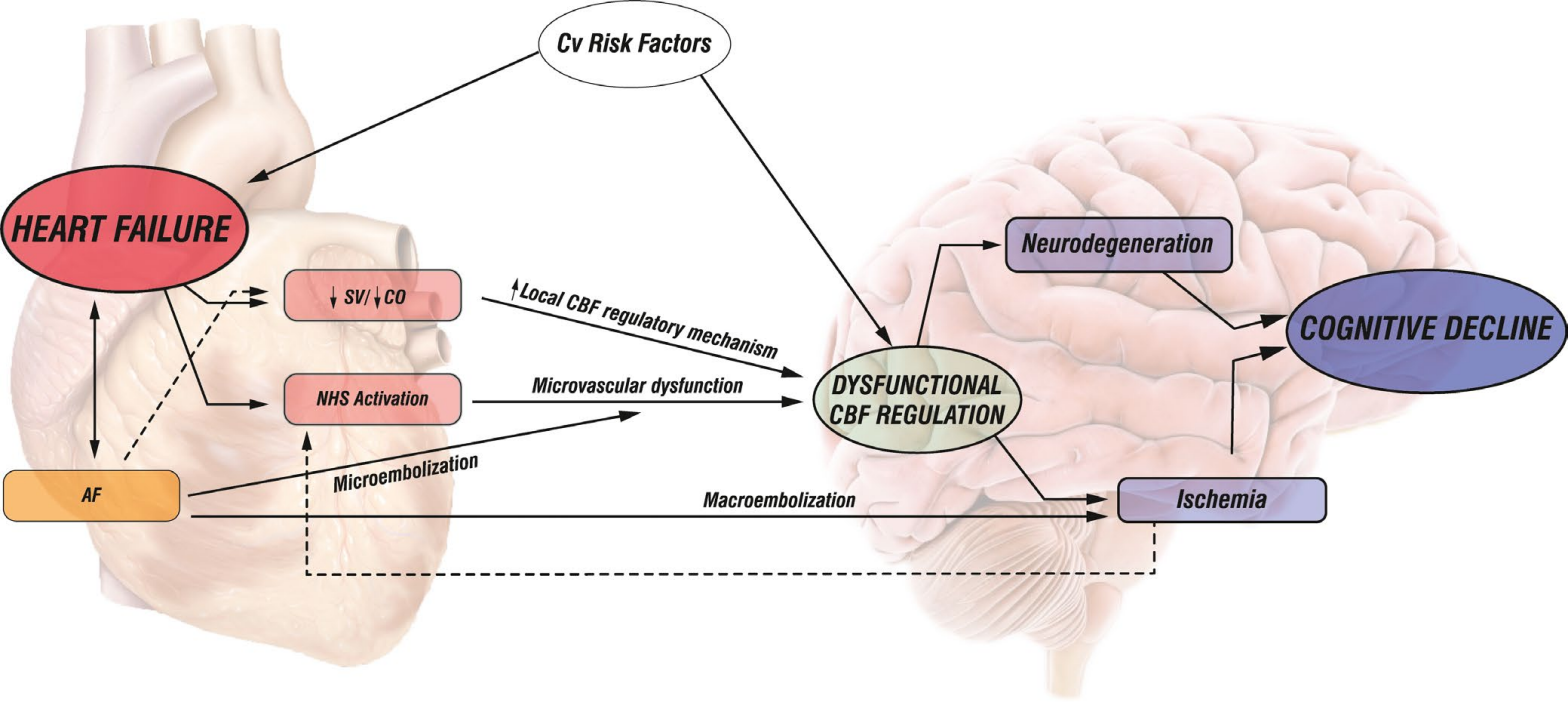
Schematic presentation of the association among impaired cardiac function in chronic heart failure, cerebral blood flow, and cognitive decline. Left: The cerebral blood flow (CBF) changes in chronic heart failure (CHF) result from cardiac dysfunction (reduced stroke volume—SV/cardiac output—CO) and activation of the neurohumoral system (NHS). Reduced SV or CO induces local CBF regulatory mechanisms, leading to arteriolar vasodilatation, compromising further vasodilatation potential, and impairing CBF regulation. NHS activation provokes vasoconstriction of cerebral vascular bed, induces structural changes in cerebral resistance vessels, and causes microvascular dysfunction leading to impaired CBF regulation. Atrial fibrillation (AF) as a specific condition may lead to CBF impairment through mechanisms activated in CHF, through microembolic events causing microvascular dysfunction and impairing CBF regulation, or through macroembolic events leading to cerebral ischemia. Right: Cognitive decline (CD) in CHF results from dysfunctional CBF regulation causing hypoperfusion and ischemia of critical brain areas or from microvascular dysfunction due to cardiovascular risk factors associated with neurodegenerative disorders. Stroke volume (SV); cardiac output (cardiac output); neurohumoral System (NHS); atrial fibrillation (AF)

In 1977, an article published in the Lancet proposed the name cardiogenic dementia for the CD observed following recurrent episodes of cardiac arrhythmias or heart disease (Dementia, 1977) The risk of CD is four times higher in CHF patients compared to matched controls without CHF (Sauve et al., 2009). Various aspects of cognitive impairment are found in patients with CHF, including the impairment of learning and working memory, attention, executive function, psychomotor speed, while the language domain and visuospatial ability are less affected (Kim & Kim, 2015). Current studies report that CD in CHF nonlinearly correlates to cardiac output and LVEF, with poorer cognitive performance when LVEF is lower than 30% (Zuccala et al., 1997). Kresge et al. (2018) were among the first to suggest that compromised global longitudinal strain, a sensitive marker of systolic function, relates to poorer episodic memory and language performance among older adults, even when LVEF is preserved. One of the proposed mechanisms underlying CD in CHF is chronic regional hypoperfusion of the brain tissue leading to atrophy in critical areas such as medial temporal lobe, which is known for having poor collateral blood flow (Vogels et al., 2007). Additionally, neuroimaging studies have suggested that subcortical white matter hyperintensities are independently related to decreased heart function (Jefferson et al., 2007; Raiha et al., 1993). The influence of cardiac dysfunction on CD is further supported by the fact that cognitive function improves after cardiac resynchronization therapy, (Hoth et al., 2010). left ventricular assist device implantation, (Zimpfer et al., 2006) and heart transplantation (Bornstein et al., 1995; Gruhn et al., 2001). Nevertheless, patients with CHF usually exert several cardiovascular risk factors for systemic as well as cerebral microvascular disease. Impairment of cerebral microvasculature plays an important role in CD and is being studied intensively as a mechanism in neurodegenerative dementias (Iadecola, 2017). Due to the complexity of CBF regulation, however, it is very challenging to perform the clinical studies necessary to clarify to what extent either cardiac dysfunction or microvascular disease contributes to CD in CHF patients. Additionally, the coexistence of both could have a nonlinear deleterious effect. Postulated mechanisms interconnecting cardiac dysfunction, CBF, and CD are summarized in Figure 2.

## 6 POTENTIAL ROLE OF CBF EVALUATION IN CHF PATIENTS

Current guidelines suggest Mini–Mental Test and the Montreal Cognitive Test to evaluate cognitive function in CHF (Ponikowski et al., 2016). Recently, a more detailed diagnostic strategy was proposed including further neurological assessment and imaging following an abnormal initial cognitive evaluation (Havakuk et al., 2017). It has been implied that cognitive impairment in CHF patients as revealed by neuropsychological testing is a consequence of impaired CBF (Bornstein et al., 1995).

It is fair to assume that patients who are at risk of CD due to CHF could be identified through CBF evaluation even before neuropsychological testing reveals cognitive impairment. Various nuclear, magnetic resonance, and sonographic imaging methods have been used to evaluate CBF in CHF; (Caldas et al., 2017; Choi et al., 2006; Erkelens et al., 2017; Georgiadis et al., 2000; Gruhn et al., 2001; Kim et al., 2012; Loncar et al., 2011; Roy et al., 2017; Serber et al., 2014; Smith et al., 2020; Song et al., 2019; Woo et al., 2009; Xie et al., 2005) however, most recent studies have found transcranial Doppler ultrasound (TCD) as a simple, noninvasive, readily available technique, which allows real‐time monitoring of functional changes and provides relevant data on impaired CBF regulatory mechanisms (Aires et al., 2020; Fabjan et al., 2015; Junejo et al., 2020). Impairment of cerebrovascular reactivity, cerebral autoregulation, and neurovascular coupling was found in various populations of CHF patients proposing TCD as a possible diagnostic tool to screen CHF patients for subtle CBF changes, which might reflect heart failure‐induced brain injury (Aires et al., 2020; Caldas et al., 2017; Erkelens et al., 2017; Georgiadis et al., 2000; Serber et al., 2014; Xie et al., 2005). However, further research including neuropsychological testing along with TCD evaluation of CBF regulatory mechanisms in CHF patients is mandatory to confirm the association between impaired CBF, as a measurement of end‐organ perfusion failure, and CD.

## 7 CONCLUDING REMARKS

The review of the contemporary literature supports the thesis that cardiac dysfunction leads to CBF impairment and predisposes to CD. It appears that the contributions to CBF impairment in such setting are multifactorial. An important role could be attributed to chronically activated neurohumoral mechanisms in CHF patients, including increased sympathetic activity, which is known to affect CBF predominantly in pathological conditions. With its vasoconstrictive effects, it could compromise CBF regulation and incline CHF patients to cerebral hypoperfusion. Microvascular damage due to cardiovascular risk factors in CHF patients could further potentiate CD in CHF. Areas with lack of collateral blood flow or increased density of perivascular sympathetic nerves, such as the medial temporal lobes and deep brain white matter, are predominantly prone to chronic global cerebral hypoperfusion. Therefore, injury in these critical brain regions is expected to be found in CHF patients, leading to CD. Understanding and identifying the basic neuropathophysiological changes involved could help with developing methods for early recognition of patients at risk, followed by institution of therapeutic actions to decrease CD in CHF patients.

## CONFLICT OF INTEREST

None to declare.

## AUTHOR CONTRIBUTIONS

All authors have made substantial contributions to the conception and design of the manuscript, acquisition of data, analysis and interpretation of data, drafting and critical revision of the article and have approved the final version to be submitted.

### PEER REVIEW

The peer review history for this article is available at https://publons.com/publon/10.1002/brb3.2176.
